# Data-driven prognostic features of cognitive trajectories in patients with amnestic mild cognitive impairments

**DOI:** 10.1186/s13195-018-0462-z

**Published:** 2019-01-22

**Authors:** Yeo Jin Kim, Seong-Kyoung Cho, Hee Jin Kim, Jin San Lee, Juyoun Lee, Young Kyoung Jang, Jacob W. Vogel, Duk L. Na, Changsoo Kim, Sang Won Seo

**Affiliations:** 1 Department of Neurology, Chuncheon Sacred Heart Hospital, Hallym University College of Medicine, Chuncheon, Korea; 2 Department of Neurology, Samsung Medical Center, Sungkyunkwan University School of Medicine, 50 Ilwon-dong, Kangnam-ku, Seoul, 135-710 Republic of Korea; 3 Neuroscience Center, Samsung Medical Center, Seoul, Korea; 4 Institute of Human Complexity and Systems Science, Yonsei University, Seoul, Korea; 5 Department of Neurology, Kyung Hee University Hospital, Seoul, Korea; 6 Department of Neurology, Chungnam National University Hospital, Daejeon, Korea; 7 Montreal Neurological Institute, McGill University, Montrèal, Quebec Canada; 8 Department of Health Sciences and Technology, SAIHST, Sungkyunkwan University, Seoul, Korea; 9 Department of Preventive Medicine, Yonsei University College of Medicine, 50 Yonsei-ro, Seodaemun-gu, Seoul, 120-752 Republic of Korea; 10 Department of Clinical Research Design and Evaluation, SAIHST, Sungkyunkwan University, Seoul, Korea

**Keywords:** Amnestic mild cognitive impairment, Cognitive trajectory, Data-driven

## Abstract

**Background:**

Although amnestic mild cognitive impairment (aMCI) is generally considered to be a prodromal stage of Alzheimer’s disease, patients with aMCI show heterogeneous patterns of progression. Moreover, there are few studies investigating data-driven cognitive trajectory in aMCI. We therefore classified patients with aMCI based on their cognitive trajectory, measured by clinical dementia rating sum of boxes (CDR-SOB). Then, we compared the clinical and neuroimaging features among groups classified by cognitive trajectory.

**Methods:**

We retrospectively recruited 278 patients with aMCI who underwent three or more timepoints of neuropsychological testing. They also had magnetic resonance imaging (MRI) including structured three-dimensional volume images. Cortical thickness was measured using surface-based methods. We performed trajectory analyses to classify our aMCI patients according to their progression and investigate their cognitive trajectory using CDR-SOB.

**Results:**

Trajectory analyses showed that patients with aMCI were divided into three groups: stable (61.8%), slow decliner (31.7%), and fast decliner (6.5%). Changes throughout a mean follow-up duration of 3.7 years in the CDR-SOB for the subgroups of stable/slow/fast decliners were 1.3-, 6.4-, and 12-point increases, respectively. Decliners were older and carried apolipoprotein E4 (APOE4) genotypes more frequently than stable patients. Compared with the stable group, decliners showed a higher frequency of aMCI patients with both visual and verbal memory dysfunction, late stage aMCI, and multiple domain dysfunction. In addition, compared with the stable group, the slow decliners showed cortical thinning predominantly in bilateral parietotemporal areas, while the fast decliners showed cortical thinning predominantly in bilateral frontotemporal areas. Both decliner groups showed worse cognitive function in attention, language, visuospatial, memory, and frontal/executive domains than the stable group.

**Conclusions:**

Our data-driven trajectory analysis provides new insights into heterogeneous cognitive trajectories of aMCI and further suggests that baseline clinical and neuroimaging profiles might predict aMCI patients with poor prognosis.

**Electronic supplementary material:**

The online version of this article (10.1186/s13195-018-0462-z) contains supplementary material, which is available to authorized users.

## Background

Mild cognitive impairment (MCI) refers to an intermediate state between normal aging and dementia with cognitive dysfunction but no functional impairment. In particular, MCI with memory deficits defined as amnestic MCI (aMCI) is considered to be a prodromal state of Alzheimer’s disease (AD) [1]. However, the clinical outcomes of aMCI patients are heterogeneous, as some patients quickly progress to AD dementia while others remain stable or even revert to normal cognition [2–4]. Identification of poor prognostic factors would therefore be important for patient counseling, risk stratification, and management.

Previous studies have shown that several factors affect the frequency of conversion to dementia or progression of aMCI [2, 5, 6]. These studies have shown that aMCI is more likely to progress in patients with brain atrophy, with evidence of amyloid deposition (measured using positron emission tomography (PET)), and carriage of the apolipoprotein E4 (APOE4) allele [7, 8]. Previous studies from our group also showed that the progression of aMCI to dementia depended on the modality and severity of involved memory dysfunction, and the multiplicity of impaired cognitive domains [9–13]. However, these studies were based on hypothesis-driven analysis, that is researchers classified participants into several subgroups according to their hypothesis. In contrast, the use of trajectory analysis is a data-driven classification approach. It is designed to identify clusters of individuals who have followed a similar trajectory on longitudinal performance. It would thus enable identification, summarization, and communication of complex prognostic factors in longitudinal data [14]. It would further provide distinct longitudinal trajectories by complex prognostic profiles as disease progresses over time. Indeed, a recent study from our group suggested that trajectory analysis is useful to identify prognostic profiles in patients with non-amnestic MCI [15].

In the present study, we applied trajectory analysis in aMCI patients to better predict their heterogeneous prognosis. First, we identified several distinct cognitive trajectories based on longitudinal performance as measured by the clinical dementia rating sum of boxes (CDR-SOB) score. Second, we tested our hypothesis that subgroups classified by trajectory analysis may reflect subgroups with known prognostic factors including the modality and severity of involved memory dysfunction, and the multiplicity of impaired cognitive domains suggested by previous studies based on hypothesis-driven analysis [12, 13, 16, 17]. Finally, we compared clinical features, genotypes, and cortical thickness among subgroups classified by trajectory analysis and identified which profiles might predict aMCI with poor prognosis.

## Methods

### Participants

We retrospectively recruited 278 patients who were clinically diagnosed with aMCI and underwent more than three timepoints of neuropsychological tests at Samsung Medical Center between March 2001 and May 2013. All patients fulfilled the Petersen’s criteria [18] for MCI with modifications: 1) subjective memory complaint by the patient or his/her caregiver; 2) normal general cognitive function above −1.0 standard deviation (SD) on the Korean version of the Mini-Mental State Examination (K-MMSE); 3) normal activities of daily living (ADL) as judged by both an interview with a clinician and the standardized ADL scale previously described [9]; 4) objective memory decline lower than −1.0 SD on the Seoul Verbal Learning test (SVLT) and Rey-Osterrieth Complex Figure Test (RCFT), which represent verbal memory and visual memory, respectively; and 5) not demented. No patient had a family history suggesting autosomal dominant disease. We excluded participants if they had a history of a neurological disorder, major depression disorder or other psychiatric disorders, substance abuse, or head trauma with loss of consciousness. Possible secondary causes for cognitive deficits were accounted for by acquiring a physiological test panel including complete blood count, blood chemistry, vitamin B12, folate, syphilis serology, and thyroid function. We excluded patients with other structural lesions detected on brain magnetic resonance imaging (MRI), including territorial infarction, intracranial hemorrhage, brain tumor, hydrocephalus, or severe white matter hyperintensity (WMH).

We obtained written informed consent from each participant, and the institutional review board of the Samsung Medical Center approved the study protocol.

### Neuropsychological tests

All patients underwent neuropsychological tests using the Seoul Neuropsychological Screening Battery (SNSB). This battery includes quantitative tests, including digit span (forward and backward), the Korean version of the Boston Naming Test (K-BNT), the RCFT (copying, immediate, and 20-min delayed recall, and recognition), the SVLT (three learning-free recall trials of 12 words, a 20-min delayed recall trial for these 12 items, and a recognition test), the phonemic and semantic Controlled Oral Word Association Test (COWAT), Stroop Test (word and color reading of 112 items during a 2-min period), the K-MMSE, and the clinical dementia rating (CDR) scale.

### Classification of aMCI patients

We classified patients with aMCI into several subgroups by the modality and severity of involved memory dysfunction, and the multiplicity of involved cognitive domains, based on previous studies from our group [9–13]. We classified patients with aMCI into three subgroups according to the modality of involved memory dysfunction: patients with only visual memory dysfunction (Visual-aMCI), those with only verbal memory dysfunction (Verbal-aMCI), and those with both visual and verbal memory dysfunction (Both-aMCI). Therefore, Verbal-, Visual-, and Both-aMCI patients have scores lower than −1.0 SD of age- and education-matched norms in the delayed recall item scores of verbal memory test, visual memory test, and both verbal and visual memory tests, respectively. We also classified patients with aMCI according to the severity of memory dysfunction. If patients with aMCI had scores on delayed recall tests between 1.0 and 1.5 SD below norms, they were considered to have mild memory dysfunction and classified as early stage aMCI (E-aMCI); if patients had scores lower than 1.5 SD below norms in delayed recall item scores of either verbal memory or verbal memory tests, they were considered to have severe memory dysfunction and classified as late stage aMCI (L-aMCI). Finally, patients with aMCI were classified according to the multiplicity of involved cognitive domains. Patients with isolated memory dysfunction were assigned as single-domain aMCI (Single-aMCI), and patients having memory impairment plus other cognitive deficits, such as language and visuospatial dysfunction, were assigned as multiple-domain aMCI (Multiple-aMCI).

### MRI techniques

Standardized T2, three-dimensional (3D) T1 turbo field echo, 3D fluid-attenuated inversion recovery (FLAIR), and DTI images were acquired from all applicable subjects at the Samsung Medical Center using the same 3.0-T MRI scanner (Philips 3.0 T Achieva). Detailed imaging parameters are described in Additional file 1 (Text S1).

### Cortical thickness measurement image processing

T1-weighted MR images were automatically processed using the standard Montreal Neurological Institute image processing software (CIVET) to measure cortical thickness. The software has been well validated and extensively described elsewhere including aging/atrophied brain studies [19–23]. Detailed imaging parameters are described in Additional file 1 (Text S2).

### APOE genotyping

Genomic DNA was extracted from peripheral blood leukocytes using the Wizard Genomic DNA Purification kit following the manufacturer’s instructions (Promega, Madison, WI, USA). Two single nucleotide polymorphisms (rs429358 for codon 112 and rs7412 for codon 158) in the *APOE* gene were genotyped using TaqManSNP Genotyping Assays (Applied Biosystems, Foster City, CA, USA) on a 7500 Fast Real-Time PCR System (Applied Biosystems) following the manufacturer’s instructions.

### Follow-up evaluations

Of the 278 patients who underwent baseline and two or more follow-up neuropsychological evaluations, 230 completed the first follow-up 1 year after the baseline assessment. One hundred and eighty-seven patients completed two consecutive follow-up neuropsychological evaluations annually. One hundred and forty-seven patients completed the follow-up more than three times. Seventy-four patients completed the follow-up more than four times. Forty-four patients completed the follow-up more than five times. Fifteen patients completed the follow-up more than six times. Five patients completed the follow-up more than seven times. Three patients completed the follow-up more than eight times (Additional file 1: Table S1).

### Conversion to dementia

We diagnosed dementia using criteria from the DSM-IV, which requires cognitive impairment detected by neuropsychological tests. Such cognitive impairment should be sufficient to interfere with independence in ADL. We did not use the MMSE or CDR-SOB scores as a diagnostic tool for dementia.

### Statistical analysis

We used group-based trajectory modeling to identify distinct cognitive trajectories. Group-based trajectory modeling fits a discrete, semiparametric mixture model to longitudinal data using maximum likelihood methods to estimate membership probabilities for multiple trajectories [24]. This approach groups individuals with the same cognitive progression trajectory in the same class. To investigate the cognitive trajectory, CDR-SOB was used. The Bayesian information criterion (BIC) was used to select the number of trajectories that best fit the data.

Demographic data were analyzed using one-way analysis of variance (ANOVA) for continuous variables and chi-square tests for categorical variables. To investigate the distribution of aMCI subtype, we performed chi-square tests. Furthermore, post-hoc analyses were performed using the Bonferroni’s correction for multiple comparisons. To compare baseline cognitive function, we performed an analysis of covariance (ANCOVA), including age, gender, and years of education as covariates. To investigate differences in the baseline mean cortical thickness, we performed an ANCOVA, including age, gender, years of education, and intracranial volume (ICV) as covariates.

Diffusion smoothing with a full-width half-maximum of 20 mm was used to blur each cortical thickness map, leading to an increased signal-to-noise ratio and an increased statistical power [20]. To evaluate the topography of cortical thickness related to gait disturbances, multiple linear regression analysis was performed on a vertex-by-vertex basis using the Surfstat package (http://www.math.mcgill.ca/keith/surfstat) after controlling for age, gender, years of education, and ICV. The cortical surface model contained 81,924 vertices; thus, correction for multiple comparisons was accomplished using the random field theory method [25] at a corrected probability value of 0.05. Trajectory analyses were conducted with SAS using Proc Traj procedure [24]. All other analyses were performed using PASW (version 18.0; SPSS, Chicago, IL, USA).

## Results

### Groups classified according to cognitive trajectories

**Fig. 1:**
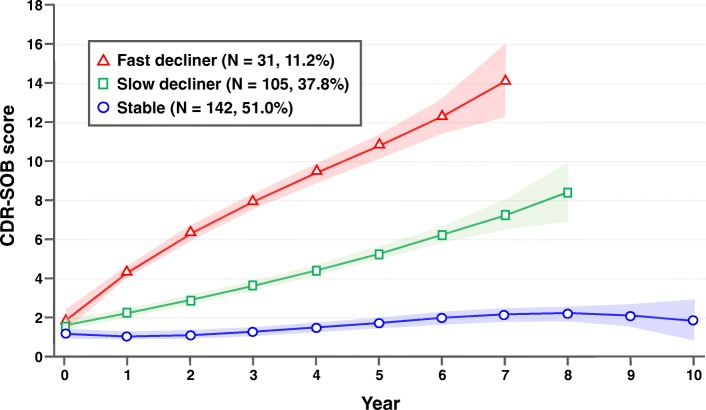
Trajectories of clinical dementia rating sum of boxes (CDR-SOB) of amnestic mild cognitive impairment

Detailed demographic and clinical characteristics of participants at baseline are presented in Table 1. To define the cognitive trajectories we evaluated models with one to five groups. According to the BIC values, the three-group model was found to be the best. The trajectories are illustrated in Fig. 1. Among 278 patients, 142 (51.0%), 105 patients (37.8%), and 31 patients (11.2%) were classified into the “stable”, “slow decliner”, and “fast decliner” trajectory groups, respectively. CDR-SOB of the stable/slow decliner/fast decliner groups were 1.01/1.61/1.98 at baseline, 1.17/3.63/8.25 at 3 years after baseline, and 1.63/5.00/10.60 at 5 years after baseline, respectively (Additional file 1: Table S2).

**Table 1:** Demographics and clinical characteristics

	Total (*n* = 278)	Stable (*n* = 142)	Slow decliner (*n* = 105)	Fast decliner (*n* = 31)	*p* value^a^
Age, years	70.9 ± 7.12	70.0 ± 6.97	71.1 ± 7.29	74.5 ± 6.17^b^	0.006
Gender, female (%)	166 (59.7)	83 (58.5)	62 (59.0)	21 (67.7)	0.624
Education, year	11.5 ± 4.90	11.5 ± 4.99	11.4 ± 4.91	11.9 ± 4.56	0.896
Vascular risk factor (%)					
Hypertension	109/244(44.6%)	52/116 (44.9%)	43/97 (44.4%)	14/31 (45.2%)	0.891
Diabetes mellitus	76/244 (31.1%)	34/116 (29.3%)	32/97 (33.0%)	10/31 (32.3%)	0.475
Hyperlipidemia	64/244 (26.2%)	33/116 (28.5%)	21/97 (21.6%)	10/31 (32.3%)	0.508
Cardiac disease	50/244 (20.5%)	21/116 (18.1%)	23/97 (23.7%)	6/31 (19.3%)	0.551
Previous stroke	8/243 (3.3%)	6/115 (5.2%)	2/97 (2.1%)	0/31 (0%)	0.374
APOE genotype (%)					
APOE e2 carrier	21 (8.4)	15 (11.6)	5 (5.5)	1 (3.6)	0.427
APOE e4 carrier	93 (37.3)	37 (28.7)	44 (48.4)^b^	11 (39.2)	0.025
Conversion to dementia (%)	124 (44.6)	10 (7.0)	83 (79.0)	31 (100)	< 0.0001
Reversion to normal (%)	31 (11.2)	31 (21.8)	0 (0)	0 (0)	< 0.0001

*APOE* apolipoprotein E

^a^Analysis of covariance (ANCOVA) or chi-square test was performed

^b^Significant difference compared with the stable group, defined as *p* < 0.05 from post-hoc analysis using Bonferroni’s correction

The mean age of patients in the fast decliner group was higher (74.5 ± 6.2, *p* = 0.006) than in the stable group (70.0 ± 7.0) (Table 1). No differences were found in gender and education among the three groups. There were also no differences in the frequency of vascular risk factors among the three groups. Patients with the *APOE* e4 allele were more frequent in the slow decliner group (48.4%) than in the stable group (28.7%).

With a mean follow-up duration of 3.7 years, 124 (44.6%) aMCI patients converted to dementia and 31 (11.2%) aMCI patients reverted to a cognitively normal state (Table 1). All fast decliners and 83 (79.0%) slow decliners converted to dementia while only 10 (7.0%) of the stable group converted to dementia. The reverters were all in the stable group.

### Distribution of aMCI subgroups

**Fig. 2:**
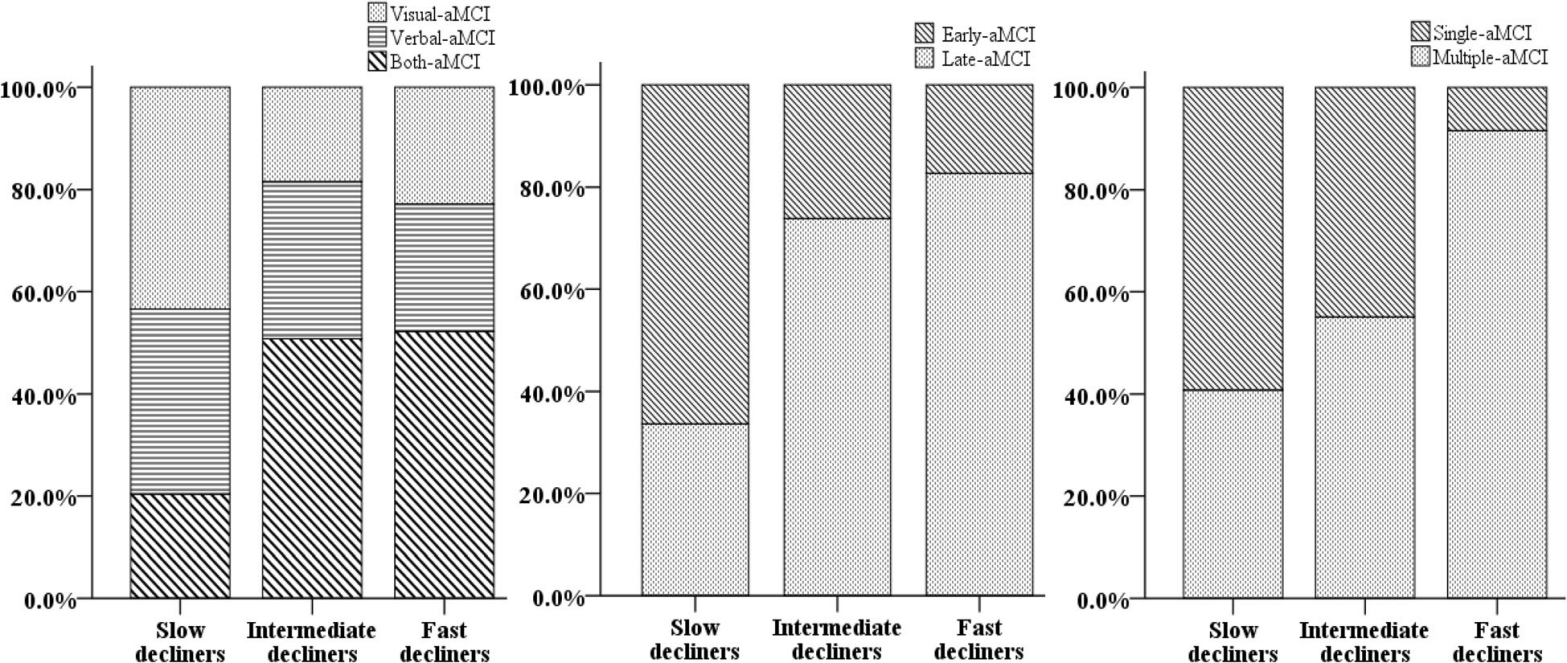
Distribution of amnestic mild cognitive impairment subgroups. aMCI amnestic mild cognitive impairment, Both-aMCI aMCI with both verbal and visual memory impairment, Early-stage aMCI aMCI with delayed recall item scores between 1.0–1.5 SD below age- and education-matched norms on both verbal and visual memory tests, Late-stage aMCI aMCI with delayed recall item scores 1.5 SD below age- and education-matched norms on either verbal or visual memory tests, Multiple-aMCI aMCI with memory and dysfunction affecting other cognitive domains, Single-aMCI aMCI with memory deficit alone, Verbal-aMCI aMCI with predominant verbal memory impairment, Visual-aMCI aMCI with predominant visual memory impairment

The distributions of aMCI subgroups are shown in Fig. 2 and Additional file 1 (Table S3). There were differences in the distribution of aMCI subgroups among the three groups. Post-hoc analyses revealed that, compared with stable aMCI, slow decliners showed a higher frequency of Both-aMCI than Visual-aMCI (*p* < 0.001) or Verbal-aMCI (*p* = 0.009) and a higher frequency of L-aMCI than E-aMCI (*p* < 0.001). Compared with the stable group, fast decliners showed a higher frequency of Both-aMCI than that of Visual-aMCI (*p* < 0.027), a higher frequency of L-aMCI than that of E-aMCI (*p* < 0.001) and a higher frequency of Multiple-aMCI than that of Single-aMCI (*p* < 0.001). Compared with slow decliners, fast decliners also showed a higher frequency of Multiple-aMCI than that of Single-aMCI (*p* = 0.045).

### Baseline neuropsychological features according to cognitive trajectories group

Compared with the stable group, the fast decliners showed lower baseline scores on K-BNT, RCFT copy, SVLT immediate and delayed recall, RCFT delayed recall, COWAT animal and supermarket, Stroop color reading, and K-MMSE (Table 2). Compared with the stable group, the slow decliners showed significantly lower scores on K-BNT, SVLT delayed recall, RCFT delayed recall, COWAT animal and supermarket, Stroop color reading, and K-MMSE. Although there was no statistically significant difference, compared with the stable group the fast decliners showed a lower score on the geriatric depression scale (GDeS) (*p* = 0.92). There were no differences in scores of neuropsychological tests between the fast decliners and the slow decliners, except for CDR-SOB.

**Table 2:** Comparison of neuropsychological tests between groups at baseline

	Stable (*n* = 142)	Slow decliner (*n* = 105)	Fast decliner (*n* = 31)
K-MMSE	27.1 ± 2.16^ab^	24.7 ± 2.98	24.4 ± 2.66
CDR SOB	1.01 ± 0.680^ab^	1.61 ± 0.766	1.98 ± 0.871^c^
Attention			
Digit span forward	5.8 ± 1.41	5.7 ± 1.52	5.6 ± 1.52
Digit span backward	3.8 ± 1.27	3.5 ± 1.00	3.4 ± 0.89
Language and related abilities			
K-BNT	42.7 ± 8.93^ab^	38.2 ± 9.60	34.6 ± 9.80
Calculation	11.0 ± 1.73	11.0 ± 1.80	11.1 ± 1.20
Ideomotor apraxia	4.6 ± 0.82^b^	4.4 ± 1.06	4.0 ± 1.37
Visuospatial function			
RCFT copy	31.0 ± 4.33^b^	29.7 ± 6.41	28.2 ± 6.64
Memory			
SVLT: immediate recall	16.1 ± 4.12^b^	14.8 ± 3.87	13.2 ± 3.73
SVLT: delayed recall	3.5 ± 2.31^ab^	1.3 ± 1.64	1.3 ± 2.29
SVLT: recognition	19.0 ± 2.75^a^	17.4 ± 2.40	18.3 ± 2.21
RCFT: immediate recall	9.0 ± 5.36	6.9 ± 10.92	6.1 ± 4.26
RCFT: delayed recall	8.6 ± 5.16^ab^	5.2 ± 3.91	5.0 ± 3.95
RCFT: recognition	18.8 ± 2.15^ab^	17.6 ± 2.24	17.2 ± 2.26
Frontal/executive function			
COWAT animal	13.5 ± 3.82^ab^	11.6 ± 4.24	10.0 ± 2.20
COWAT supermarket	14.4 ± 5.88^ab^	11.8 ± 4.72	9.6 ± 3.47
COWAT phonemic	22.4 ± 10.64	21.2 ± 10.90	18.6 ± 7.30
Stroop test: word reading	109.0 ± 8.90	107.1 ± 12.76	108.4 ± 10.71
Stroop test: color reading	73.6 ± 24.28^ab^	62.9 ± 25.21	52.7 ± 19.93
Geriatric depression scale	12.8 ± 7.25	11.9 ± 7.01	9.2 ± 5.55

Analysis of covariance (ANCOVA) was performed after adjusting for age, gender, and years of education, followed by post-hoc analysis with Bonferroni’s method. Bonferroni’s correction was also used to correct for multiple neuropsychological tests (defined as *p* < 0.05)

*CDR-SOB* clinical dementia rating sum of boxes, *COWAT* Controlled Oral Word Association Test, *K-BNT* Korean version of the Boston Naming Test, *K-MMSE* Korean version of Mini-Mental State Examination, *RCFT* Rey-Osterrieth Complex Figure Test, *SVLT* Seoul Verbal Learning Test,

^a^*p* < 0.05 with Bonferroni’s post-hoc analyses comparing the stable group and the slow decliner group

^b^*p* < 0.05 with Bonferroni’s post-hoc analyses comparing the stable group and the fast decliner group

^c^*p* < 0.05 with Bonferroni’s post-hoc analyses comparing the slow decliner group and the fast decliner group

### Baseline cortical thickness according to cognitive trajectory group

At baseline, the mean cortical thickness of the fast decliners was lower than the stable aMCI group in the frontal, temporal, and parietal regions, and the mean cortical thickness of the slow decliners was lower than the stable group for all lobes (Additional file 1: Table S4).

**Fig. 3:**
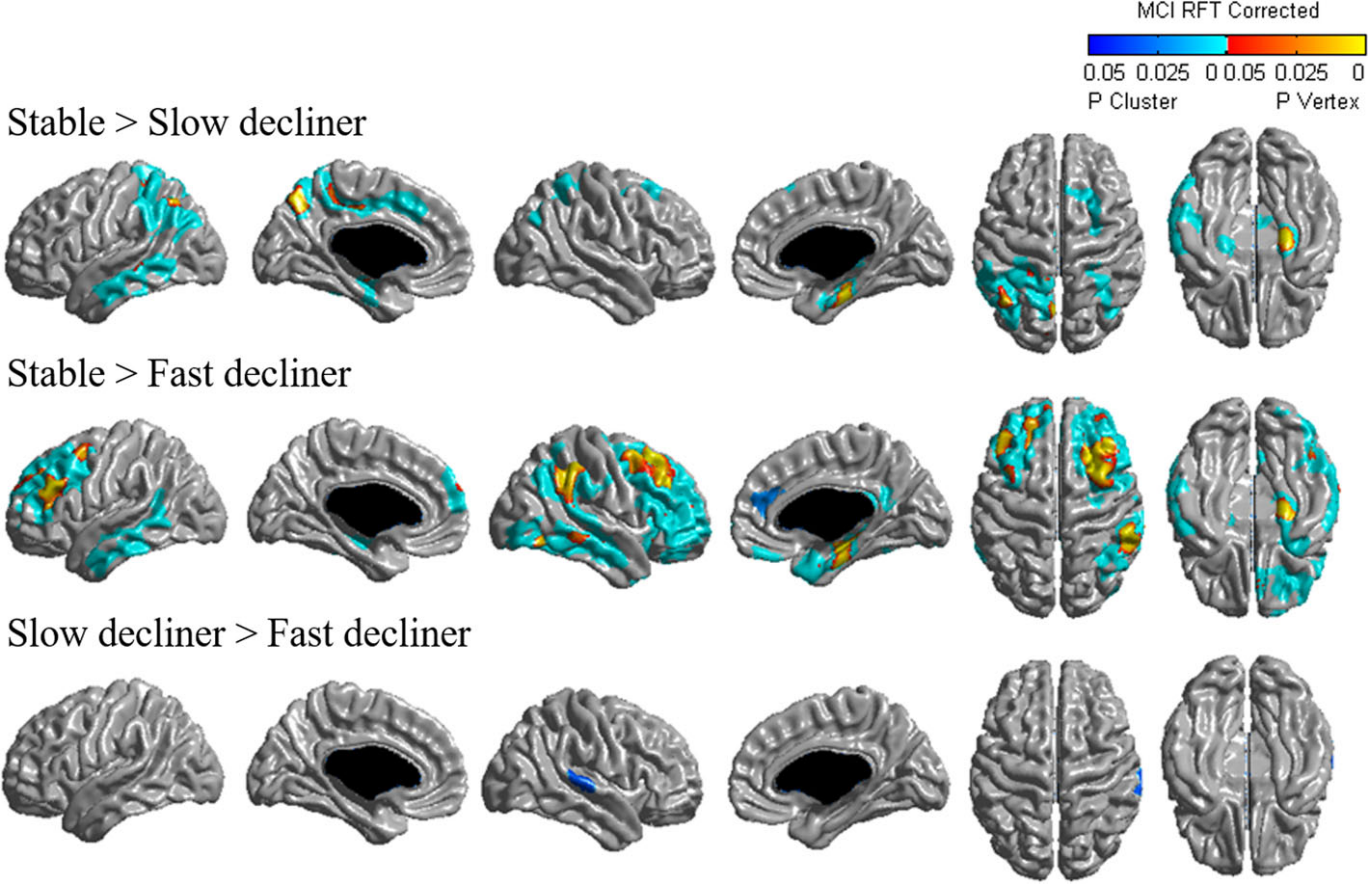
Statistical maps comparing baseline cortical thickness. Localized differences in cortical thickness between stable, slow decliners, and fast decliners were analyzed with a general linear model on a vertex-by-vertex basis after controlling for age, gender, years of education, and intracranial volume (defined as *p* < 0.05). MCI mild cognitive impairment, RFT random field theory

Cortical thinning topography at baseline is shown in Fig. 3. The fast decliners showed more cortical thinning than the stable group in the bilateral dorsolateral and medial frontal, lateral temporal, right orbitofrontal, lateral parietal, medial temporal, and lateral occipital regions. The slow decliners showed more cortical thinning in the bilateral lateral parietal, medial temporal, left medial frontal, lateral temporal, and right dorsolateral frontal regions compared with the stable group. The fast decliners showed more cortical thinning in the right superior temporal gyrus than the slow decliners.

## Discussion

In the present study, trajectory analysis of aMCI classified three distinct cognitive trajectories including stable aMCI, slow decliners, and fast decliners, which may reflect subgroups with known prognostic factors suggested by previous studies [9–11]. We also found that, compared with the stable group, the slow decliners and the fast decliners showed more extensive cortical thinning in the parietotemporal and frontotemporal regions at baseline, respectively. In addition, both types of decliners had worse baseline cognition and more domains of impaired cognition compared with the stable group. Taken together, our data-driven trajectory analysis provides new insights into heterogeneous cognitive trajectories of aMCI and further suggest that baseline clinical and neuroimaging profiles might predict aMCI patients with poor prognosis.

Our conclusion that the trajectory analysis was useful for identifying distinct trajectories of stable, slow decliner, and fast decliners is supported by the following observations. First, as we expected, all fast decliners and most of the slow decliners showed conversion to dementia while none of decliners reverted to cognitively normal. Second, subgroups classified by the trajectory analysis reflect subgroups with known prognostic factors suggested by previous studies based on hypothesis-driven analysis [2, 9–13, 16, 17, 26, 27], that is previous studies showed that, depending on the modality and severity of involved memory dysfunction and multiplicity of involved cognitive domains, some subtypes of aMCI had more cortical thinning or faster progression than other subtypes of aMCI [16]: Both-aMCI compared with Visual-aMCI [10, 12, 26]; L-aMCI compared with E-aMCI [11, 13, 27]; and Multiple-aMCI compared with Single-aMCI [2, 9, 17]. Our present study also suggested similar results, showing that Both-aMCI, L-aMCI, and Multiple-aMCI were included more frequently in the decliners than the stable group, compared with Visual-aMCI, E-aMCI, and Single-aMCI, respectively.

One major finding was that, compared with the stable group, the decliners showed more extensive cortical thinning in the parietotemporal and frontotemporal regions, respectively. This is consistent with our other finding showing that, compared with the stable group, the slow decliners and the fast decliners showed lower scores in language, memory, and frontal/executive function domains. The present finding is also consistent with our previous study based on non-amnestic MCI showing that decliners had decreased cortical thickness compared with the stable group [15]. These regions are known to be predominantly affected in AD [28]. Specifically, brain atrophy from MCI to AD starts in the medial temporal region and then spreads to the posterior temporal regions, followed by parietal regions, and finally to frontal regions [29, 30]. The temporoparietal involvement of the slow decliners might imply earlier features of AD pathology. Meanwhile, the fast decliners showed more cortical thinning in regions extending to and including the frontal lobe. Previous studies reported that frontal involvement predicted a more rapid progression of cognitive decline in aMCI [6, 31]. Our findings therefore suggest that the slow and fast decliners might represent earlier and later stages of AD-like cortical thinning patterns, respectively.

We also found that the fast decliners showed decreased cortical thickness in the right superior temporal gyrus relative to the slow decliners. When pathologic changes appear in the superior temporal gyrus, it corresponds to Braak stage V of cortical neurofibrillary pathology [32]. This means that the superior temporal gyrus is one of the areas involved in advanced stage AD. In a tau-PET study, most MCI patients were assigned to Braak stage III/IV, while tracer uptake in neocortical Braak regions (Stage V) was related to structural and cognitive markers of AD [33]. This suggests that uptake of the more advanced Braak stage regions might be more sensitive to the transition from MCI to dementia. Therefore, thinning of the right superior temporal gyrus might be associated with rapid progression of aMCI; however, more evidence is needed to confirm this result.

The strength of the trajectory analysis performed in this study is the identification of comprehensive clinical and imaging variables predicting individuals who would show rapid progression. In the present study, we found that the clinical and imaging variables such as old age, APOE4 carrier, decreased cortical thickness, and worse cognitive impairments discriminated decliners from the stable group. This finding is consistent with previous studies showing that older age at diagnosis predicted more rapid progression of aMCI or conversion to dementia [16, 34]. In addition, the APOE4 gene is the major genetic risk factor for the conversion of aMCI to AD [35], likely acting through various mechanisms including impaired amyloid clearance. Furthermore, higher CDR-SOB and decreased cortical thickness in the right superior temporal gyrus predicted rapid decliners.

Interestingly, the stable group seemed to be different from the decliner groups. A few (7.0%) in the stable group converted to dementia with a mean follow-up period of 5 years, and over 20% of those in the stable group reverted to a cognitively normal state [36]. Considering another finding that GDeS scores were higher in the stable group than in decliners, cognitive impairment in the stable group might be partially related to other causes including depression or anxiety rather than AD. Further studies with molecular biomarkers are needed.

There are several limitations to our study. First, patients were selected from a single center, and several patients withdrew during the follow-up period resulting in a relatively small number of patients therefore limiting the generalizability of the results. Second, the aMCI diagnosis was based on clinical phenotype and without pathologic confirmation, and we therefore could not identify the pathological underpinnings of the patients included in this study. Finally, and unexpectedly, 51% of aMCI patients were classified into the stable aMCI group. The proportion of stable aMCI patients seems to be higher compared with previous studies showing a stable aMCI proportion of about 40% [16, 37]. The higher proportion of stable aMCI participants in our study might be related to selection bias (i.e., survival bias in the cohort study). That is, the stable group has been followed up continuously while decliners were dropped during the follow-up period. Another potential possibility is related to the differences in study population (early stage and late stage of aMCI in our sample compared with late-stage aMCI in the previous studies) [38].

## Conclusions

In the present study, our data-driven trajectory analysis provides new insights into heterogeneous cognitive trajectories of aMCI. Our findings clearly suggest that identification of comprehensive baseline clinical and neuroimaging profiles from trajectory analysis may help to detect individuals at greatest risk for dementia and thus inform interventions.

## Additional file

Additional file 1: Supplementary texts and tables. (DOC 82 kb)

## Abbreviations

3D: Three-dimensional
AD: Alzheimer’s disease
ADL: Activities of daily living
aMCI: Amnestic mild cognitive impairment
ANCOVA: Analysis of covariance
ANOVA: Analysis of variance
APOE4: Apolipoprotein E4
BIC: Bayesian information criterion
CDR: Clinical dementia rating
CDR-SOB: Clinical dementia rating sum of boxes
COWAT: Controlled Oral Word Association Test
FLAIR: Fluid-attenuated inversion recovery
GDeS: Geriatric depression scale
ICV: Intracranial volume
K-BNT: Korean version of the Boston Naming Test
K-MMSE: Korean version of Mini-Mental State Examination
MCI: Mild cognitive impairment
MMSE: Mini-Mental State Examination
MRI: Magnetic resonance imaging
PET: Positron emission tomography
RCFT: Rey-Osterrieth Complex Figure Test
SD: Standard deviation
SNSB: Seoul Neuropsychological Screening Battery
SVLT: Seoul Verbal Learning Test
WMH: White matter hyperintensity
