# Supplementary material for: Data-driven prognostic features of cognitive trajectories in patients with amnestic mild cognitive impairments
Source: Alzheimers Res Ther. 2019 Jan 22;11:10. doi: 10.1186/s13195-018-0462-z (PMC6343354; doi:10.1186/s13195-018-0462-z)
Supplement: Supplementary file 1 — Supplementary texts and tables. (DOC 82 kb) [file 13195_2018_462_MOESM1_ESM.doc]

**Supplementary text 1**

Imaging parameters for MRI acquisition

We acquired 3D T1 turbo field echo MR images with the following imaging parameters: sagittal slice thickness, 1.0 mm, over contiguous slices with 50% overlap; no gap; repetition time (TR) of 9.9 msec; echo time (TE) of 4.6 msec; flip angle of 8°; and matrix size of 240 × 240 pixels, reconstructed to 480 × 480 over a field of view (FOV) of 240 mm. The following parameters were used for the 3D FLAIR images: axial slice thickness of 2 mm; no gap; TR of 11000 msec; TE of 125 msec; flip angle of 90°; and matrix size of 512 × 512 pixels. In whole-brain DT-MRI examination, sets of axial diffusion-weighted single-shot echo-planar images were collected with the following parameters: 128×128 acquisition matrix, 1.72×1.72×2 mm^3^ voxel size; 70 axial slices; 22 × 22 cm^2^ field of view; TE 60ms, TR 7696ms; flip angle 90◦; slice gap 0mm; b-factor of 600smm^−2^. Diffusion-weighted images were acquired in 45 different directions using the baseline image without weighting [0,0,0]. All axial sections were acquired parallel to the anterior commissure-posterior commissure.

**Supplementary text 2**

Cortical thickness measurement image processing

Native MRI images were first registered into a standardized stereotaxic space using an affine transformation.^1^ Non-uniformity artifacts were corrected using N3 algorithm, and the registered and corrected volumes were classified as white matter, gray matter, cerebrospinal fluid, and background by using an artificial neural net classifier.^2, 3^ The surfaces of inner and outer cortex were automatically extracted by deforming a spherical mesh onto the gray/white boundary in each hemisphere, by using the Constrained Laplacian-Based Automated Segmentation with Proximities algorithm, which have also been well-validated and extensively described elsewhere.^4, 5^

Cortical thickness was calculated as the Euclidean distance between the linked vertices of the inner and outer surfaces, after applying an inverse transformation matrix to cortical surfaces and reconstructing them in the native space.^5, 6^ To control for brain size, we computed intracranial volume (ICV) using classified tissue information and skull mask which was acquired from T1-weighted image.^7^ ICV is defined as the total volume of gray matter (GM), white matter (WM), and cerebrospinal fluid (CSF) with consideration of voxel dimension. Classified GM, WM, CSF, and background within the mask were transformed back into individual native space.

To compare the thicknesses of corresponding regions among the subjects, the thicknesses were spatially registered on an unbiased iterative group template by matching sulcal folding pattern using surface-based registration that performs sphere-to-sphere warping. ^8, 9^ For global and lobar regional analysis, we used the lobe-parcellated group template that had been divided into frontal, temporal, parietal, and occipital lobes by using SUMA ([http://afni.nimh.nih.gov](http://afni.nimh.nih.gov/)) from a previous study.^10^ Averaged values for the thickness of the whole vertex in each hemisphere and lobar region were used for the global analysis.

References

1. Collins DL, Neelin P, Peters TM, Evans AC. Automatic 3D intersubject registration of MR volumetric data

in standardized Talairach space. J Comput Assist Tomogr 1994;18:192-205.

2. Sled JG, Zijdenbos AP, Evans AC. A nonparametric method for automatic correction of intensity

nonuniformity in MRI data. IEEE transactions on medical imaging 1998;17:87-97.

3. Zijdenbos AP, Evans AC, Riahi F, Sled JG, Chui J, V. K. Automatic quantification of multiple sclerosis

lesion volume using streotaxic space. Proc, 4th Intl Conf on Visualization in BioMed Computing VBC,

Lecture Notes in Computer Science 1996;1131:439-448.

4. MacDonald D, Kabani N, Avis D, Evans AC. Automated 3-D extraction of inner and outer surfaces of

cerebral cortex from MRI. NeuroImage 2000;12:340-356.

5. Kim JS, Singh V, Lee JK, et al. Automated 3-D extraction and evaluation of the inner and outer cortical

surfaces using a Laplacian map and partial volume effect classification. NeuroImage 2005;27:210-221.

6. Im K, Lee JM, Lee J, et al. Gender difference analysis of cortical thickness in healthy young adults with

surface-based methods. NeuroImage 2006;31:31-38.

7. Smith SM. Fast robust automated brain extraction. Hum Brain Mapp 2002;17:143-155.

8. Lyttelton O, Boucher M, Robbins S, Evans A. An unbiased iterative group registration template for cortical

surface analysis. NeuroImage 2007;34:1535-1544.

9. Robbins S, Evans AC, Collins DL, Whitesides S. Tuning and comparing spatial normalization methods.

10.1186/s13195-018-0462-z

Med Image Anal 2004;8:311-323.

10. Im K, Lee JM, Lyttelton O, Kim SH, Evans AC, Kim SI. Brain size and cortical structure in the adult human

brain. Cereb Cortex 2008;18:2181-2191.

Supplementary Table S1. The year of assessment at each follow-up

|  | 0 year | 1year | 2years | 3years | 4years | 5years | 6years | 7years | 8years | 9years | 10years | Total |
| --- | --- | --- | --- | --- | --- | --- | --- | --- | --- | --- | --- | --- |
| Baseline | 278 |  |  |  |  |  |  |  |  |  |  | 278 |
| 1^st^ follow up |  | 230 | 37 | 6 | 2 | 0 | 0 | 2 | 0 | 1 | 0 | 278 |
| 2^nd^ follow up |  |  | 187 | 62 | 20 | 6 | 0 | 0 | 2 | 0 | 1 | 278 |
| 3^rd^ follow up |  |  |  | 86 | 41 | 12 | 4 | 2 | 0 | 1 | 1 | 147 |
| 4^th^ follow up |  |  |  |  | 42 | 23 | 6 | 3 | 0 | 0 | 0 | 74 |
| 5^th^ follow up |  |  |  |  |  | 23 | 16 | 2 | 2 | 1 | 0 | 44 |
| 6^th^ follow up |  |  |  |  |  |  | 6 | 4 | 5 | 0 | 0 | 15 |
| 7^th^ follow up |  |  |  |  |  |  |  | 1 | 2 | 2 | 0 | 5 |
| 8^th^ follow up |  |  |  |  |  |  |  |  | 0 | 2 | 1 | 3 |
| 9^th^ follow up |  |  |  |  |  |  |  |  |  | 0 | 1 | 1 |

Each cell represents the number of patients

Supplementary Table S2. Mean CDR-SOB at each follow up

|  | Stable | Slow decliner | Fast decliner |
| --- | --- | --- | --- |
| Baseline | 1.01 ± 0.680 | 1.61 ± 0.766 | 1.98 ± 0.871 |
| 1 year | 1.00 ± 0.588 | 2.17 ± 0.948 | 3.82 ± 1.264 |
| 2 years | 0.98 ± 0.586 | 2.98 ± 1.150 | 6.93 ± 2.699 |
| 3 years | 1.17 ± 0.737 | 3.63 ± 1.443 | 8.25 ± 2.179 |
| 4 years | 1.42 ± 0.909 | 4.60 ± 1.616 | 8.63 ± 2.546 |
| 5 years | 1.63 ± 1.077 | 5.00 ± 1.159 | 10.60 ± 1.817 |
| 6 years | 2.05 ± 1.371 | 5.89 ± 1.537 | 14.00 |
| 7 years | 2.30 ± 1.513 | 8.50 ± 2.291 | 14.00 |
| 8 years | 2.25 ± 1.318 | 8.00 |  |

CDR-SOB, Clinical Dementia Rating sum-of-boxes

Supplementary Table S3. Frequency of aMCI subgroups between trajectory groups

|  | Total  (278) | Stable  (142) | Slow  decliner (105) | Fast  decliner (31) | *p* value |
| --- | --- | --- | --- | --- | --- |
| Modality |  |  |  |  | <0.0001 |
| Visual (%) | 61 (21.9) | 46 (32.4) | 11 (10.5) | 4 (12.9) |  |
| Verbal (%) | 70 (25.2) | 44 (31.0) | 21 (20.0) | 5 (16.1) |  |
| Both (%) | 147 (52.9) | 52 (36.6) | 73 (69.5) | 22 (71.0) |  |
| Severity |  |  |  |  | <0.0001 |
| Early stage (%) | 69 (24.8) | 56 (39.4) | 11 (10.5) | 2 (6.5) |  |
| Late stage (%) | 209 (75.2) | 86 (60.6) | 94 (89.5) | 29 (93.5) |  |
| Multiplicity |  |  |  |  | 0.001 |
| Single (%) | 74 (26.6) | 49 (34.5) | 24 (22.9) | 1 (3.2) |  |
| Multiple (%) | 204 (73.4) | 93 (65.5) | 81 (77.1) | 30 (96.8) |  |

aMCI, amnestic mild cognitive impairment; Verbal-aMCI, aMCI with predominant verbal memory impairment; Visual-aMCI, aMCI with predominant visual memory impairment; Both-aMCI, aMCI with both verbal and visual memory impairment, Early-stage aMCI, aMCI with delayed recall item scores between 1.0-1.5 SD below age- and education-matched norms on both verbal and visual memory tests; Late-stage aMCI, aMCI with delayed recall item scores 1.5 SD below age- and education-matched norms on either verbal or visual memory tests; Single-aMCI, aMCI with memory deficit alone; Multiple-aMCI, aMCI with memory and dysfunction affecting other cognitive domains; SD, standard deviation

The Chi-square test was performed, followed by *post hoc* analysis with Bonferroni’s correction (defined as p value < 0.05)

Supplementary Table S4. Comparison of cortical thickness at baseline

|  | Stable (n=109) | Slow decliner (n=92) | Fast decliner (n=28) |
| --- | --- | --- | --- |
| Total | 2.9794 ± 0.13783*† | 2.8962 ± 0.11904 | 2.8497 ± 0.11121 |
| Frontal lobe | 3.0469 ± 0.14580*† | 2.9757 ± 0.12585 | 2.9073 ± 0.12679 |
| Temporal lobe | 3.1537 ± 0.15486*† | 3.0573 ± 0.13063 | 2.9862 ± 0.14702 |
| Parietal lobe | 2.8296 ± 0.15072*† | 2.7368 ± 0.13255 | 2.7273 ± 0.12135 |
| Occipital lobe | 2.6086 ± 0.13284* | 2.5308 ± 0.14122 | 2.5263 ± 0.13324 |

* Comparison of stable and slow decliners

† Comparison of stable and Slow decliners

ANCOVA was performed after adjusting for age, gender, years of education, and intracranial volume followed by *post hoc* analysis with the Bonferroni method (defined as p value < 0.05)
